# mTOR pathway: A current, up-to-date mini-review (Review)

**DOI:** 10.3892/ol.2014.2608

**Published:** 2014-10-10

**Authors:** PAUL ZAROGOULIDIS, SOFIA LAMPAKI, J. FRANCIS TURNER, HAIDONG HUANG, STYLIANOS KAKOLYRIS, KONSTANTINOS SYRIGOS, KONSTANTINOS ZAROGOULIDIS

**Affiliations:** 1 Pulmonary Department-Oncology Unit, G. Papanikolaou General Hospital, Aristotle University of Thessaloniki, Thessaloniki 57010, Greece; 2 Interventional Pulmonary and Critical Care Medicine, Western Regional Medical Center, Goodyear, Arizona 85338, USA; 3 Department of Respiratory Diseases Shanghai Hospital, Second Military University Hospital, Shanghai 210000, P.R. China; 4 Oncology Department, University General Hospital of Alexandroupolis, Democritus University of Thrace, Alexandroupolis 68100, Greece; 5 Oncology Department, Sotiria General Hospital, University of Athens, Athens 11527, Greece

**Keywords:** mTOR, lung cancer, targeted therapy

## Abstract

Mammalian target of rapamycin (mTOR) is a protein serine/threonine kinase that was initially identified as the cellular target of rapamycin. This kinase regulates cell growth, proliferation, motility and survival, as well as the gene transcription and protein synthesis that are activated in response to hormones, growth factors and nutrients. Results from preclinical studies have indicated that factors antagonizing the mTOR pathway exert an antitumor effect on lung cancer. Furthermore, primary clinical trials of mTOR inhibitors have demonstrated that the inhibitors may be effective against lung carcinoma. The present study explores the association between mTOR and lung carcinogenesis and describes the clinical trials of mTOR inhibitors.

## 1. Introduction

Mammalian target of rapamycin (mTOR) is a component of the phosphatidylinositol 3-kinase (PI3K) cell survival pathway that monitors the availability of nutrients, mitogenic signals and cellular energy and oxygen levels, and therefore is significant in the regulation of cell growth and proliferation (1). Abnormal activation of the PI3K pathway is considered to be involved in numerous cancers, and increased activation of this pathway is often associated with resistance to cancer therapies (2,3). mTOR acts upstream and downstream of Akt, operating at a key junction in the PI3K pathway (4). mTOR can form two different multiprotein complexes, mTORC1 and mTORC2, that regulate the protein synthesis necessary for cell growth and proliferation (4–6). Targeted molecular therapy has an established benefit when combined with platinum-based chemotherapy in phase III randomized trials of patients with metastatic non-small cell lung cancer (NSCLC) (7). Agents targeting vascular endothelial growth factor and epidermal growth factor receptor (EGFR) mimic several novel targeted approaches that improve survival in patients with lung cancer. Tyrosine kinase (TK) inhibitors, including erlotinib and gefitinib, block the intracellular TK domain of EGFR and subsequently cause a blockade of downstream signaling (8). During the process of identifying novel agents, studies have focused on characterizing relevant signaling pathways downstream from surface receptors. A previous study has reported that mTOR is a crucial component of such pathways (9).

## 2. The mammalian target of rapamycin pathway

Ligand-bound activation of one of the transmembrane receptors leads to the activation of PI3K (10,11). PI3K subsequently phosphorylates Akt, which is dephosphorylated by PTEN (12,13). Loss of PTEN is connected with a diminished prognosis in NSCLC, likely due to the enhanced downstream signaling of the PI3K/Akt/mTOR pathway (14). The two mTOR complexes, mTORC1 and mTORC2, are each involved in cell growth (15,16). mTORC1, which consists of mTOR, Raptor, GβL (mammalian lethal with SEC13 protein 8) and domain-containing mTOR-interacting protein (DEPTOR), is partially inhibited by rapamycin (17); it unifies multiple signals that indicate the availability of growth factors, nutrients and energy in order to promote cellular growth and catabolic processes during stress (18,19). Growth factors and hormones, such as insulin, use Akt to signal mTORC1, which inactivates tuberous sclerosis complex 2 to prevent inhibition of mTORC1 (20). Active mTORC1 exerts numerous downstream biological effects, including the translation of mRNA by phosphorylating downstream targets, such as 4E-BP1 and p70 S6 kinase, the suppression of autophagy through Atg13 and ULK1, ribosome biogenesis, and activation of transcription that leads to increased mitochondrial activity or adipogenesis (21–23). mTORC2, which consists of mTOR, Rictor, GβL, Sin1, PRR5/Protor-1 and DEPTOR, promotes cell survival through the activation of Akt (24,25). mTORC2 regulates cytoskeletal dynamics, and ion transport and growth by activating PKCα and phosphorylating SGK1, respectively (26–28). mTOR is a downstream target of EGFR and MET signaling, and is therefore considered to be a therapeutically attractive target for the treatment of various types of cancer.

## 3. Preclinical data

Numerous preclinical studies have suggested that mTOR and associated kinases are significant in the development of lung cancer. In a previous study, a spectrum of murine lung tissue was assessed, including normal lung, atypical alveolar hyperplasia, adenoma and adenocarcinoma tissues obtained from K-ras mice (29). Immunohistochemical staining for p-S6 was performed, revealing an elevated level of p-S6 present at each stage of the progression of malignancy. Subsequent studies have suggested that treatment with mTOR inhibitors leads to a reduction in the size and number of early neoplastic lesions. Other studies have investigated the activity of mTOR itself and the upstream regulator Akt (30). Using tissue microarray (TMA) constructs that included >100 specimens from patients with NSCLC, positive staining for mTOR was exhibited in ~74% of tumors. The literature contains data indicating the efficacy of TKIs when EGFR mutations are present, and there are also studies that have reported an involvement of K-ras mutations in conferring resistance to EGFR-targeting monoclonal antibodies (31–35). In an analysis of TMA constructs containing 37 lung tumors, mTOR activation was identified in 89% of tumors bearing K-ras or EGFR mutations (36). Another preclinical study examined the effect of a combined blockade of MEK and mTOR (37) as MEK activation intersects with mTOR activation at a number of levels. There have been numerous reports of preclinical data that supports the combination of erlotinib with an mTOR inhibitor (38–45). In one study, 22 cell lines from four tumor types, NSCLC, breast, pancreatic and colon tumors, were assessed and it was revealed that mutations in PTEN, EGFR, PI3K and K-ras were present in each cell line (46).

## 4. Clinical trials

Numerous mTOR inhibitors have been revealed to provide antitumor effects in lung cancer. A two-part phase I study assessed the antitumor activity, toxicity and pharmacokinetics of everolimus, administered weekly in 5–30 mg doses, at increased weekly doses of 50–70 mg and daily administration. In total, 92 patients participated in this study (47), 12 of whom suffered from NSCLC and two from SCLC. Compensatory tolerance of everolimus doses of ≤70 mg per week or 10 mg daily was observed. Toxicities, including stomatitis and fatigue, were observed in one patient, dosed at 50 mg per week and hyperglycemia was observed in another patient, dosed at 10 mg per day. Partial responses were observed in four patients and four patients exhibited progression-free survival (PFS) of ≥6 months.

Following this trial, an additional phase II trial enrolled patients with NSCLC into two arms: Arm 1 comprised patients that exhibited a performance status (PS) <2 and had failed <2 cycles with platinum based therapy and arm 2 comprised patients that had undergone <2 cycles of platinum based therapy in combination with an EGFR antagonist. These patients were administered everolimus at a dose of 10 mg daily. Partial response (PR) was reported in 5.3% of arm 1 patients and 2.8% of arm 2 patients. The median PFS was 11.3 weeks for arm 1 and 9.7 weeks for arm 2 patients. The observed toxicities were stomatitis, cough and dyspnea (48).

Another phase II study investigated patients with SCLC. The patients were free from brain metastasis, had relapsed following one or two regimens and exhibited a PS <2. Everolimus was administered until the disease progressed or until the onset of unacceptable toxicity. Of the 16 patients, three exhibited stable disease and the remaining patients exhibited progression. Everolimus was well tolerated, however, the efficacy of the drug was low (49). An additional phase II study assessed the effectiveness of temsirolimus alone in patients with SCLC, following treatment with four or six cycles of platinum-based therapy with etoposide or irinitecan (50). Temsirolimus was intravenously administered weekly at a dose of 25 mg (arm A) and 250 mg (arm B) until disease progression was observed. In 85 patients, the overall survival for arm A was 6.6 months and 9.5 months for arm B.

Deferolimus, a non-prodrug rapamycin analogue, was administered in a phase I trial. In total, 32 patients were administered with 3–28 mg of deferolimus daily. The maximum tolerated dose was 18.75 mg. Of the five patients with NSCLC included in the study, only one exhibited PR (51). An additional phase I study assessed treatment with gefitinib and everolimus in patients with progressive NSCLC. Gefitinib was administered at a dose of 250 mg daily and everolimus was administered at a dose of 5–10 mg daily. Of the eight patients evaluated, two exhibited PR (52). Following this, a phase II trial was designed for patients who were previous smokers with stage IIIB/IV NSCLC (53). The study comprised untreated patients (arm A) and patients who had previously received a platinating agent and docetaxel (arm B). PR was observed in 17% (arm B) of the patients. The toxicities identified were diarrhea, mucositis and rash. In another phase I trial, the combination of everolimus with erlotinib was investigated. This cohort consisted of patients with advanced NSCLC who had previously received two chemotherapy regimens and had an ECOG PS<2. Patients were excluded from the trial if they had been previously treated with an EGFR inhibitor. A standard six and six dose escalation design was administered with daily doses of 2.5 and 5 mg and weekly doses of 30 and 40 mg of everolimus, combined with 75, 100 and 100 mg of erlotinib daily. However, the response data of this trial were moderate (54).

## 5. Conclusion

All of the aforementioned preclinical and clinical trials revealed significant positive results for the use of mTOR antagonists in lung cancer. mTOR expression may be upregulated by numerous mechanisms in the pathogenesis of lung cancer. Furthermore, preclinical data suggests that this class of mTOR pathway antagonists exert an antitumor effect in lung cancer therapy. Consistent with this, initial clinical trials of mTOR inhibitors suggest that they are effective in NSCLC and small cell lung carcinoma therapy. Several phase II and III trials are currently in progress. These additional clinical trials are required to assess the efficacy of mTOR inhibitors as targeted therapy for NSCLC.

## Figures and Tables

**Figure 1:**
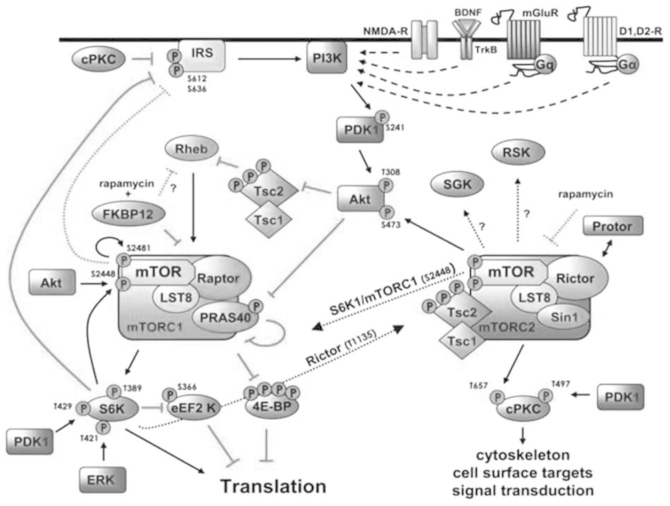
Activation of mammalian target of rapamycin occurs through a complex signaling cascade. mTOR, mammalian target of rapamycin; PI3K, phosphatidylinositol 3-kinase.
